# Leveraging Deep Learning in Real-Time Intelligent Bladder Tumor Detection During Cystoscopy: A Diagnostic Study

**DOI:** 10.1245/s10434-025-17015-3

**Published:** 2025-03-06

**Authors:** Zixing Ye, Yingjie Li, Yujiao Sun, Chengqing He, Guanglin He, Zhigang Ji

**Affiliations:** 1 Department of Urology, Peking Union Medical College Hospital, Chinese Academy of Medical Sciences and Peking Union Medical College, Beijing, China; 2 Chinese Academy of Medical Sciences and Peking Union Medical College, Beijing, China; 3 Hangzhou Hikvision Digital Technology Co., Ltd., Hangzhou, China

**Keywords:** Bladder lesion, Cystoscopy, Transurethral resection of bladder tumor, Artificial intelligence, Deep learning

## Abstract

**Background:**

Accurate detection of bladder lesions during cystoscopy is crucial for early tumor diagnosis and recurrence monitoring. However, conventional visual inspection methods have low and inconsistent detection rates. This study aimed to evaluate the effectiveness of the HRNetV2 deep learning model for intelligent bladder lesion detection, focusing on its performance at different image resolutions.

**Patients and Methods:**

We recruited 94 patients undergoing cystoscopy or transurethral resection of bladder tumor and collected 102 white-light cystoscopy videos between July 2022 and July 2023. Frames containing suspected bladder lesions were manually annotated. HRNetV2, a semantic segmentation model, was used to identify tumor-related morphological features. Images were categorized as high- or low-resolution, and the dataset was split into training and test sets (4:1 ratio). Diagnostic performance was assessed using sensitivity, precision, and mean Dice (mDice) score.

**Results:**

A total of 33,657 frames were annotated. The overall sensitivity and precision for the test set were 91.6% and 91.3%, respectively, with an mDice score of 80.3%. In the high-resolution group, sensitivity and precision were 94.8% and 94.4%, while in the low-resolution group, they were 75.6% and 74.8%. The mDice scores for high- and low-resolution images were 84.7% and 56.6%, respectively.

**Conclusions:**

HRNetV2 demonstrated excellent performance, particularly with high-resolution images, offering high sensitivity and precision in bladder lesion detection. The results suggest that HRNetV2 has significant potential to improve detection accuracy in clinical practice. Future work should focus on optimizing the model further and testing it with larger, multicenter datasets.

**Supplementary Information:**

The online version contains supplementary material available at 10.1245/s10434-025-17015-3.

Endoscopic procedures (cystoscopy and transurethral resection of bladder tumor [TURBT]) are essential investigation methods for the identification and treatment of bladder lesions, particularly in patients with bladder cancer who require long-term follow-up. However, modern cystoscopy continues to rely on the conventional white light technique developed eight decades ago. This reliance can lead to problems, including subjective interpretation and variability in diagnostic outcomes among examiners with different expertise levels or during repeat cystoscopies by the same examiner; this can potentially result in misdiagnosis, the missing of early stage lesions, and the complication of objective comparison of cystoscopy findings across reports.^1^ Previous studies have reported that approximately 10–20% of bladder cancers are missed during cystoscopy,^2^ with reported sensitivity and specificity of 68–100% and 57–97%, respectively.^3,4^ Consequently, improving cystoscopy accuracy is essential for detecting lesions and early tumor recurrence. Although blue light and narrow band imaging have transformed endoscopic imaging and significantly improved bladder tumor detection rates,^5,6^ subjective identification of cystoscopy images with the naked eye is inevitable, and complex procedures limit their further application.

Image recognition and segmentation algorithms based on deep learning have emerged as powerful artificial intelligence technologies widely used in medical practice, including tumor pathological classification and lesion detection using endoscopy.^7^ The convolutional neural network (CNN), a subset of deep learning algorithms, is notable for its robust performance in image classification.^8^ Bladder lesion detection using CNN algorithms is accurate, objective, reproducible, and automatic.^9^ Shkolyar et al. introduced CystoNet, a CNN-based automated image-processing platform for bladder cancer diagnosis, which demonstrated excellent diagnostic performance^10^ and has been used for prospective tests.^11^ Furthermore, Takuya Iwaki’s deep learning system was more accurate in diagnosing Hunner-type interstitial cystitis than urologists.^12^ Hence, deep learning-based cystoscopy augmentation is expected to significantly promote the clinical practice of comprehensive bladder lesion detection and individualized management.^11^

Image semantic segmentation has evolved from traditional methods based on shallow features to deep learning techniques, including fully convolutional network (FCN) and its extensions.^13^ A high-resolution network (HRNet) was developed in 2019^14^ and subsequently modified to HRNetV2, a superior version of HRNet.^15^ HRNetV2 significantly enhances contextual feature extraction and improves semantic segmentation accuracy by resolving downsampling effects and closing semantic gaps in information integration.^16^ Moreover, HRNetV2 maintains high-resolution representations throughout the process, without the conventional bottleneck design.^17^ Kinshuk et al. reported that HRNetV2 exhibited superior mask detection performance in social distancing calculation tasks.^18^ However, no study has been conducted on the HRNetV2 model application in medical image semantic segmentation.

In this study, we utilized the HRNetV2 to detect real-time intelligent bladder lesions during cystoscopy. This study aimed to investigate the potential of deep learning technology in assisting examiners in rapidly and accurately identifying bladder lesions that the naked eye may miss and laying the foundation for further pathological and diagnostic applications.

## Patients and Methods

### Data Collection

This study was approved by the Ethics Committee of Peking Union Medical College Hospital, Chinese Academy of Medical Sciences (I-23PJ1996). The study adhered to the ethical standards of the Helsinki Declaration. We recruited 94 patients who underwent cystoscopy or TURBT at Peking Union Medical College Hospital and collected 102 white light videos between 1 July 2022 and 1 July 2023. All patients provided written informed consent and complete baseline data. Two urologists confirmed that all the collected videos contained bladder lesions and were randomly divided into training and test sets in a 4:1 ratio. All images containing bladder lesions were extracted from video segments at fixed intervals, and manual annotation of bladder lesion outlines was performed frame by frame. The final labeled results were reviewed and verified independently by two different urologists.

### Semantic Segmentation Method

**Fig. 1:**
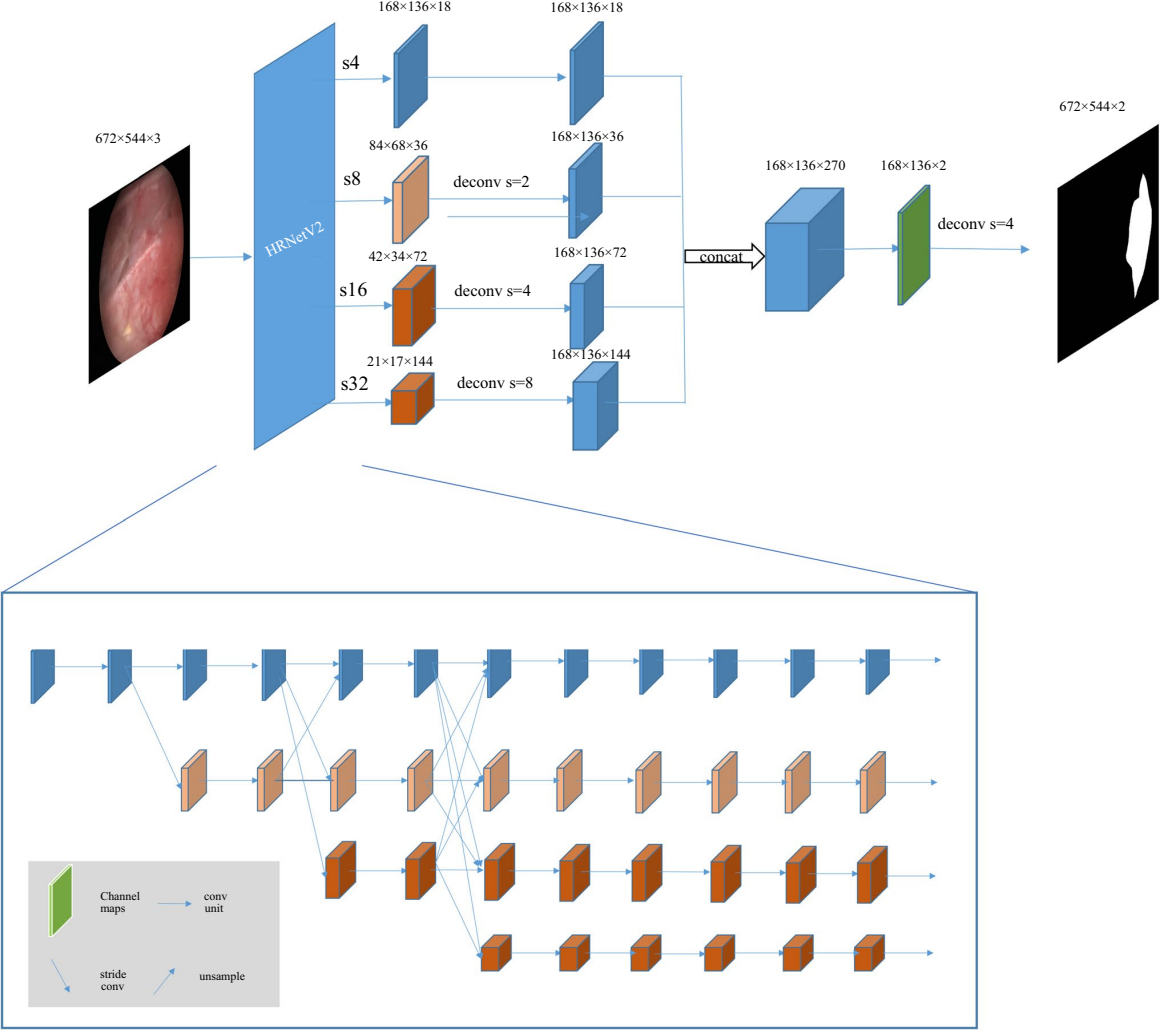
Architecture of HRNetV2 network

The semantic segmentation method was utilized for bladder lesion detection. We utilized HRNetV2, a modified version of HRNet,^17^ as the encoder. FCN^19^ served as the decoder in the FCN_HRNetV2 segmentation network formation (Fig. 1). In this configuration, the encoder maintains high resolution, allowing for the extraction of detailed positional features. The HRNetV2 produces feature maps at four scales, which were subsequently unified to a consistent resolution using deconvolution. The decoder restores the feature maps to their original image size.

### Data Preprocessing and Training

The original input data resolutions for bladder tumor recognition were 544 × 672, 480 × 720, and 1080 × 1920. Before inputting the data into the network, images of these sizes were standardized using red, green, and blue (RGB) channels, with the mean and variance values of (123.675, 116.28, and 103.53) and (57.12, 57.12, and 57.12), respectively. After normalization, the images were fed into the network, which produced output sizes that matched the original input dimensions. After segmentation, a simple post-processing step was implemented using a 29 × 29 kernel. This involved an initial morphological opening operation to remove edge spikes in the predicted regions and a closing operation to remove small fragmented areas.

We utilized a batch size 16 on a single NVIDIA Tesla V100 Tensor Core graphics processing unit (GPU) during training. The backbone employed an ImageNet pretrained model optimized using the stochastic gradient descent (SGD) optimizer with an initial learning rate of 0.01. We used a polynomial learning rate decay strategy, ensuring a minimum learning rate of 1 × 10^−4^. The training involved cropping the images to 512 × 512 dimensions. To ensure stability in bladder lesion detection despite differences in size and angles during cystoscopy, we employed image augmentation techniques, including flipping and contrast and brightness enhancement. In addition, we utilized a feature pyramid structure to integrate feature maps of different scales, enhancing the performance of the model across diverse scenarios. The final model was achieved after 80,000 iterations. We employed a single V100 tensor core GPU for inference.

### Model Evaluation

The performance of the segmentation model was evaluated using intersection over union (IOU), with a 0.1 IOU threshold chosen to prioritize bladder lesion detection over precise localization. The diagnostic performance was further evaluated on the basis of sensitivity and precision in the test set at the specified IOU threshold. In addition, the mean Dice (mDice) score was used as the evaluation metric for the detection performance of the model, characterizing its ability to detect bladder tumors across different resolution levels.

## Results

In this study, 33,657 frames were manually annotated, outlining 37,947 targets. Among these, 75 patients contributed 30,103 targets (26,654 frames) to the training set, while 19 patients contributed 7,844 targets (7003 frames) to the test set. Overall, the diagnostic sensitivity of the neural network model in the test set was 91.6%, with a precision of 91.3% (Table 1). The video increment experiment demonstrated a steady improvement in model performance with increasing data (Table 2).

**Table 1:** Diagnostic performance of HRNetV2 at different resolution levels

	Training set	Test set		
		High-resolution group	Low-resolution group	Total
Patients	75	–	–	19
Videos	82	–	–	20
Frames	26,654	5897	1106	7003
Ground truth	30,103	6522	1322	7844
Sensitivity	–	94.8%	75.6%	91.6%
Precision	–	94.4%	74.8%	91.3%
mDice score	–	84.7%	56.6%	80.3%

**Table 2:** Results of video increment experiment

Videos trained	Frames trained	Sensitivity (%)	Precision (%)
15	5421	76.7	39.2
68	22,270	91.1	90.1
82	29,528	91.6	91.3

**Fig. 2:**
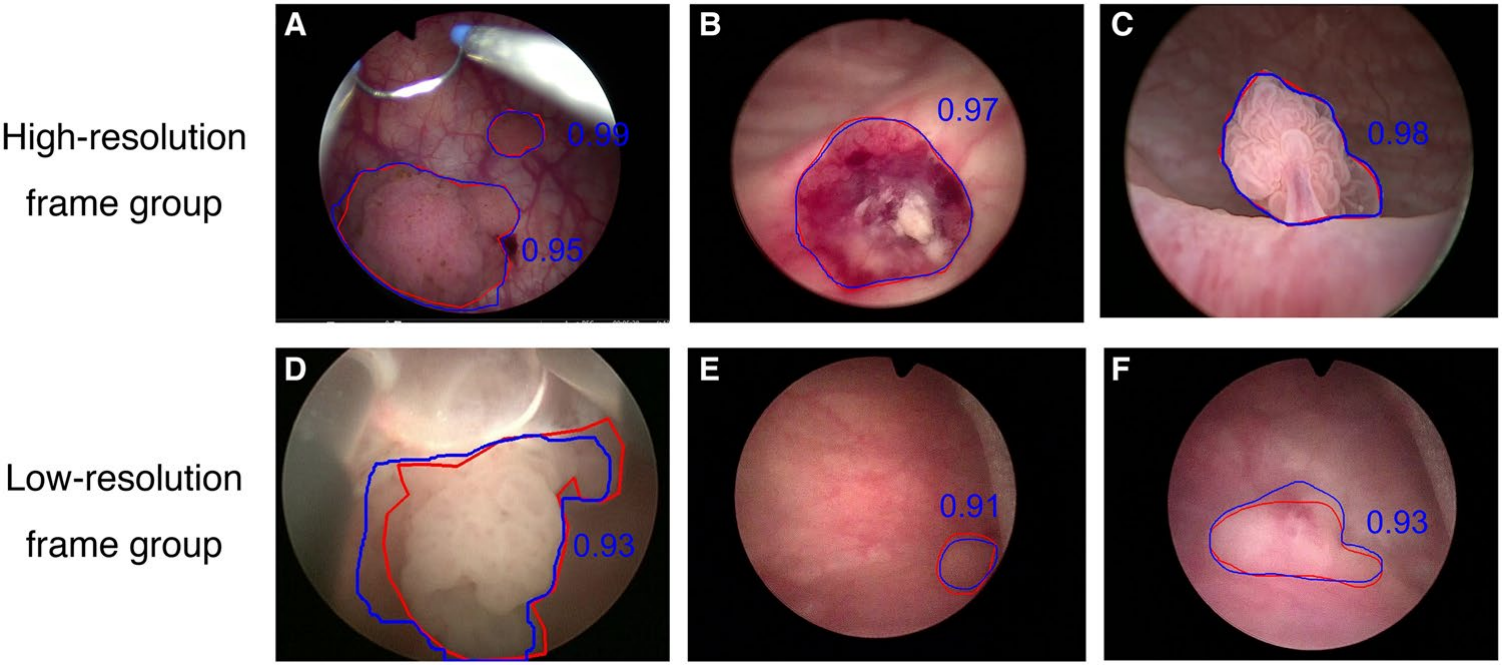
Representative frames of high-resolution frame group and low-resolution frame group; the numbers represent IOU values

Frames in the test set were artificially categorized into high-resolution and low-resolution frame groups on the basis of lesion clarity and contrast with surrounding tissues to better align with clinical practice and facilitate subsequent work. Frames in the high-resolution group typically exhibited clear morphology and a relatively intact structure, resembling aquatic grass or cauliflower. The low-resolution frames were characterized by an incomplete appearance due to excision, an unclear feature from the camera distance, or turbid fluid interference (Fig. 2). The sensitivity and precision in the high-resolution group were 94.8% and 94.4%, respectively, compared with 75.6% and 74.8% in the low-resolution group. The mDice scores for these groups were 84.7% and 56.6%, respectively, with an overall mDice of 80.3% (Table 1). Supplementary files 1 and 2 show representative demos demonstrating real-time bladder lesion detection at different resolutions.

We investigated the impact of ground truth (GT) area proportion on diagnostic performance. Table 3 shows that the model performed poorly when the GT area proportion was small (0–0.02), with a recall rate of 0.564. Suboptimal performance was observed when the GT area approached half of the image (0.5–0.7). However, this conclusion may lack reliability owing to the limited number of targets in this category.

**Table 3:** Diagnostic performance under different GT area proportions

Proportion	Recall	GT	Correct predictions	Total predictions	Sensitivity (%)	Precision (%)
0–0.02	564	1000	745	1230	56.4	60.6
0.02–0.05	1384	1484	1209	1276	93.3	94.7
0.05–0.1	1208	1256	1090	1124	96.2	97.0
0.1–0.2	2307	2330	2144	2195	99.0	97.7
0.2–0.3	1000	1007	1045	1067	99.3	97.9
0.3–0.4	411	414	507	518	99.3	97.9
0.4–0.5	187	187	333	349	100	95.4
0.5–0.6	93	114	154	159	81.6	96.9
0.6–0.7	27	49	24	26	55.1	92.3
0.7–1.0	3	3	4	4	100	100

**Fig. 3:**
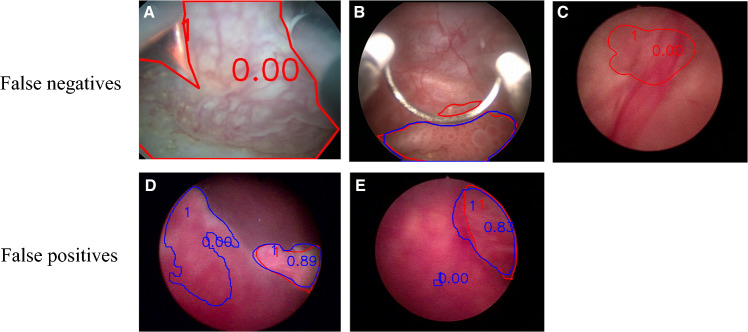
Analysis of false negatives and false positives; the main reasons for false negatives are (**A**) close targets, (**B**) small size, and (**C**) atypical features; the main reasons for false positives are abnormal (**D**) texture and (**E**) small targets mistaken for lesions; red lines indicate manual annotations, and blue lines represent algorithm predictions; the numbers represent IOU value

In addition to assessing diagnostic and detection performance, we analyzed the impact of image quality and potential factors contributing to false diagnoses. False positives were primarily due to abnormal mucosal texture features and the misidentification of small targets as lesions. False negatives were attributed mainly to atypical target features, relatively small target sizes, and targets that were too close to the observation point (Fig. 3).

## Discussion

Bladder tumors are typically numerous, immobile, and frequently recurring. Papillary tumors are easy to identify. However, they are difficult to classify, whereas flat tumors, including carcinoma in situ (CIS), are difficult to detect and can grow rapidly without intervention.^20^ Therefore, conducting a thorough and systematic examination of all potential lesions is essential when planning future follow-ups or determining the need for adjuvant treatments.^21^ However, experienced medical personnel may overlook bladder tumors because of their inherent subjectivity and the possibility of interference from operational or observational factors. We employed the recently proposed HRNetV2 neural network model for real-time bladder lesion detection and achieved excellent diagnostic and detection performance. This was evidenced by high sensitivity, precision, and mDice scores.

Deep learning techniques for computer vision include semantic segmentation and object detection. Unlike object detection, which provides object location, semantic segmentation precisely delineates the spatial boundaries of targets, providing more information for classification and subgroup analysis.^22^ Our study aimed to achieve real-time and comprehensive detection of all suspicious lesions within the bladder cavity using deep learning methods. This will serve as the foundation for subsequent lesion evaluation and therapeutic guidance by testing the diagnostic and detection performance of the deep learning model. Therefore, employing the semantic segmentation model HRNetV2 and selecting patients with suspected bladder lesions rather than those with bladder tumors is consistent with the aim of this study.

Numerous studies have investigated the use of CNNs in the intelligent diagnosis of intravesical lesions. For example, Ikeda et al. conducted a pilot study with 1671 normal and 431 tumor images to diagnose bladder cancer. The deep learning model GoogLeNet achieved a sensitivity of 89.7% and a specificity of 94.0%.^23^ Refining algorithms led to superior performance. Zhang et al. investigated attention mechanisms in bladder tumor segmentation using the classical U-Net model, and the new network achieved an improved mDice score of 82.7%.^24^ Strategies, including expanding sample datasets, contribute to more stable diagnosis performance. Wu et al. developed the cystoscopy artificial intelligence diagnostic system (CAIDS) framework using a pyramid scene parsing network. They collected > 69,000 cystoscopy images from > 10,000 patients to train the model for tumor diagnosis, and the accuracies of different external validation sets all surpassed 97%. Our study demonstrated that the stability of the model improved as the sample size increased. Additionally, CAIDS was found to diagnose carcinoma CIS more accurately than urologists.^25^ A previous study compared eight deep neural networks for the semantic segmentation of cystoscopy images and found that the Pyramid Attention Network (PAN) model performed better than the others.^22^ Algorithm enhancement and dataset expansion are crucial for the further advancement of deep learning for bladder lesion detection based on the methodologies employed in various studies.

Cystoscopy uses water for observations, unlike most medical endoscopes, including gastrointestinal endoscopy, which uses air to conduct light. Therefore, cloudy urine, blood mist, and light scattering, commonly encountered in clinical practice, can affect the imaging quality. Low-visibility medical images obscure essential disease details and decrease diagnostic accuracy, which are major causes of missed and false detections of deep learning models. We performed subgroup analysis to address the influence of image quality on detection performance. We demonstrated that HRNetV2 exhibits superior sensitivity and precision for frames with higher resolution than other models, reaching 94.8% and 94.4%, respectively, with a mDice score of 84.7%. Considering that the overall sensitivity and precision exceeded 90% and the overall mDice score exceeded 80%, it is evident that, despite cystoscopy being conducted in a liquid environment, the HRNetV2 model can achieve satisfactory detection performance similar to that of endoscopy and even surpass it in cases where visibility is high. However, there is still a considerable gap in detecting low-resolution frames compared with other published models. The diagnostic sensitivity and precision for low-resolution bladder lesions are 75.6% and 74.8%, respectively, with a mDice score of 56.6%. Therefore, maintaining good transparency of the bladder cavity fluid is essential to ensuring the effectiveness of deep learning.

We investigated the observational and operational factors influencing poor visibility, as it is the most common cause of abnormal detections. We prioritized missed detections above false positives, since missed detections can lead to more severe adverse consequences. Reduced visibility can occur when the lens is too close or far from the target, resulting in an out-of-focus image. Moving the lens can partially improve this. Another cause of undiagnosed lesions is the indistinct differentiation between bladder tumor features and normal mucosal tissue, especially for flat lesions. In these cases, the lack of multiangle observations makes it difficult for model identification. Further improvement requires additional training data with multiangle and dynamic observation views to improve performance. Morphological similarity to trained features is the leading cause of false positive detection. This highlights the need for more data to improve the segmentation effect of bladder lesions. A low GT ratio can lead to false negative and false positive detections, as indicated by our GT area ratio test. Real-time panoramic image stitching for cystoscopy may be a useful solution to this problem. Further investigation into image enhancement techniques, including noise filtering and image dehazing,^21^ could improve performance.

This study has some limitations. First, it is a single-center study with limited datasets, which affects the detection efficiency of the model. To address this, we plan to collect more data and conduct multicenter studies. Second, the imbalanced distribution of various pathological types within the datasets, particularly the lack of samples of CIS, prevented tumor classification. This highlights the need to augment the dataset with various pathology types. Third, although HRNetV2 shows satisfactory results, its performance compared with other models may not be optimal. Future research should focus on improving algorithms based on HRNetV2 for better bladder tumor detection.

## Conclusions

Our study highlights the potential of integrating the HRNetV2 algorithm for real-time intraoperative detection of bladder lesions. This method shows promise in improving the accuracy and efficiency of bladder lesion diagnosis in clinical practice. Continued refinement and optimization of our method will provide valuable insights for future research in the field of medical image processing and lay the foundation for subsequent analyses, including pathology classification.

## Supplementary Information

Below is the link to the electronic supplementary material.

Supplementary file1 (MP4 8844 KB)

Supplementary file2 (MP4 8858 KB)

## Data Availability

The data that support the findings of this study are available from the corresponding author, Z.J., upon reasonable request.
